# Association between eating disorders, suicide and depressive symptoms in undergraduate students of health-related courses

**DOI:** 10.31744/einstein_journal/2020AO4908

**Published:** 2019-12-10

**Authors:** Vanigleidson Silva do Nascimento, Alisson Vinicius dos Santos, Suammy Barros Arruda, Gabriela Avelino da Silva, Joanna D'arc de Souza Cintra, Tiago Coimbra Costa Pinto, Rosana Christine Cavalcanti Ximenes

**Affiliations:** 1 Universidade Federal de Pernambuco, Recife, PE, Brazil

**Keywords:** Feeding and eating disorders, Depression, Suicide, Students, health occupations

## Abstract

**Objective:**

To identify symptoms of eating disorders and potential associations with risk of suicide and depressive symptoms in undergraduate students of health-related courses.

**Methods:**

A cross-sectional study involving 271 students. The following instruments were used to identify symptoms of eating disorders: Eating Attitudes Test-26 and Bulimic Investigatory Test of Edinburgh. The Hamilton Depression Rating Scale and the Mini International Neuropsychiatric Interview were used to screen for depressive symptoms and risk of suicide, respectively. Participants answered a questionnaire aimed to collect biodemographic data for economic classification of the sample.

**Results:**

Symptoms of eating disorders and bulimia nervosa were detected in 7.4% and 29.1% of students, respectively. Approximately 17.3% of students had symptoms of major depression, and 13.6% were at risk of suicide to some extent; risk of suicide was thought to be low in 7.4%, moderate in 0.7% and high in 5.5% of students in this subset. The risk of eating disorder development was correlated with the risk of suicide (p<0.001).

**Conclusion:**

Undergraduate students at risk of developing eating disorders, or with symptoms suggestive of depression, are more prone to commit suicide.

## INTRODUCTION

Imposition of beauty standards and stigmatization may lead to rejection of obese or overweight individuals, giving rise to feelings of contempt and dissatisfaction with one's own body and fear of gaining weight.^(^^1^^)^ Eating behavior changes and weight control practices are associated with personal dissatisfaction and low self-esteem, which may culminate in weight loss.^(^^2^^)^

Media plays a major role in advocating a lean or even skinny body type as the ideal of beauty and health, with increasing value attributed to body weight and shape, particularly among women. People are therefore led to adopt practices with severe negative impacts on health, such as excess physical exercise and extreme diets aimed to achieve an ideal body.^(^^3^^)^

Growing body dissatisfaction and suicide rates over the last few years have led to the inclusion of eating disorders in the list of psychiatric disorders. Before, there was no pre-established cause.^(^^3^^–^^5^^)^

Eating disorders are severe, multifactorial psychiatric disorders characterized by altered eating behaviors and comprising two major subtypes: anorexia nervosa and bulimia nervosa.^(^^1^^,^^3^^,^^4^^)^ These disorders are associated with high mortality rates, disability, physical and psychological morbidity, and poorer quality of life.^(^^6^^,^^7^^)^ Huge attention on the part of public health policies is required, given victims tend to hide the disease and not to seek professional help.^(^^1^^)^

Physical and psychological changes typical of adolescence may trigger eating disorders development from this age, primarily anorexia nervosa and bulimia nervosa, as the cognitive maturity required for adaptation to physiological changes is lacking.^(^^8^^)^ Many diagnosed cases are closely related to high mortality rates, with 15% of patients progressing to death.^(^^2^^,^^7^^)^ Among other factors, eating disorders may contribute to risk of suicide,^(^^8^^)^ one of the major causes of morbidity and mortality among adolescents and young adults due to increased adoption of risk behaviors in these stages of life.^(^^9^^)^

Several associations involving suicide risk have been reported, such as family history of suicide, substance use, maltreatment of children and psychiatric disorders, with special emphasis on mood disorders, eating disorders (mainly anorexia nervosa and bulimia nervosa) and symptoms of major depression. However, of all psychiatric disorders, eating disorders seem to be the most associated with suicide risk.^(^^10^^)^ Depression prevalence is higher in people suffering from eating disorders as compared to the general population.^(^^11^^)^

Between 15% and 25% of undergraduate students are estimated to experience some sort of psychiatric disorder over the course of graduation, particularly in health-related areas.^(^^9^^,^^11^^)^ However, epidemiological studies focusing on this subset are scarce. Eating disorders may be associated with depression among undergraduate students, with increased risk of suicide in this population.

## OBJECTIVE

To identify eating disorder symptoms and determine potential associations with suicide risk and depressive symptoms in students taking undergraduate courses in health-related areas.

## METHODS

A cross-sectional epidemiological study conducted at a public university in the interior of the state of Pernambuco. The sample comprised male and female students aged 18 years or more and enrolled in undergraduate health-related courses. On-campus population comprised 1,525 students. The following courses were on offer: Nursing, Nutrition, licentiate degree in Biological Sciences, licentiate and bachelor degree in Physical Education and Collective Health. The small number of students taking the latter course precluded its inclusion in this analysis.

Epi Info™ was used to calculate the size of a representative sample of on-campus students. Baseline prevalence derived form a pilot study was also used. This sample was selected to check the suitability of proposed methods and instruments, train researchers and determine a baseline prevalence for final sample size calculation. Assessments carried out during this phase were aimed at testing and were not included in the main study. Hence, based on the 33.1% estimated prevalence of eating disorders symptoms derived from a 30-student pilot study with 5.0% error and 95.0% reliability, a sample comprising 278 students was obtained. However, 7 students dropped out, leaving a final sample of 271.

Students’ names were randomly drawn out of the university attendance list. Selected students were invited to participate in the study while in the classroom. Whenever students were absent or refused to participate, the next student on the list was invited.

Data were collected in the classroom during break time, over the course of 3 months. Undergraduates participated in data collection during their shift. The socioeconomic profile of the selected sample was defined using a sociodemographic questionnaire.^(^^12^^)^ The following data were collected: age, sex, number of siblings and birth order, number of rooms in the household and number of people living in the same household. The questionnaire also comprised economic classification data, according to *Critérios de Classificação Econômica do Brasil* 2015 [Brazilian Economic Classification Criteria], published by *Associação Brasileira das Empresas de Pesquisa* (ABEP) [Brazilian Association of Research Companies]. The following socioeconomic strata are described in this classification, according to average household income: A, R$ 20.888,00 or higher; B1, from R$ 9.254,00; B2, above R$ 4.852,00; C1, above R$ 2.705,00; C2, above R$ 1.625,00; D, above R$ 768,00 and E, below the latter figure.

Eating disorder symptom identification was based on the EAT-26 test (Eating Attitudes Test-26).^(^^13^^,^^14^^)^ Items divided into three Likert-type scales and comprising six response alternatives, each are rated zero to 3 according to participants’ choice. Rates per item are then summed up and a final score obtained. Participants scoring ≥21 were considered to be at risk of eating disorders development, whereas those scoring zero to 20 were considered to be risk-free. The test is not diagnostic but allows for detection of clinical cases in high risk populations and identification of individuals with abnormal concerns regarding eating and body weight.^(^^2^^,^^8^^)^

Bulimia nervosa occurrence and severity were assessed using the Bulimic Investigatory Test of Edinburgh (BITE).^(^^15^^,^^16^^)^ This test comprises a symptom (30 yes/no items scored zero to 30) and a severity (three dimensional items) scale. Both scores may be summed up to obtain a total score. High symptom scale scores (≥20) may suggest severely disrupted eating behavior and compulsive overeating; medium scores (10 to 19) suggest unusual eating behavior calling for clinical interview assessment, and low scores (below 10) are within normal limits. As regards the severity scale, scores ≥5 and ≥10 are rated clinically significant and highly severe, respectively.

The Brazilian version of the Mini International Neuropsychiatric Interview (MINI) 5.0.0, Module C – Risk of Suicide consists of a 19-module interview interrogating 17 axis 1 disorders listed in Diagnostic and Statistical Manual of Mental Disorders, Fourth Edition™ (DSM-IV™), risk of suicide and anti-social personality disorder.^(^^17^^)^ Only module C – Risk of Suicide was evaluated in this study. Students were rated as follows: score zero, “no risk of suicide”; score 1 to 5, “low risk of suicide”; score 6 to 9, “moderate risk of suicide”; score 10 or higher, “high risk of suicide”.

The self-assessment questionnaire of the Hamilton Depression Rating Scale (HAM-D) developed at the University of London based on the Hamilton Depression Scale^(^^18^^)^ has been translated into Portuguese^(^^19^^)^ and validated for the Brazilian population. Using a cutoff value of 10, data obtained via the HAM-D were rather precise as regards the identification of depressed patients from normal controls.^(^^18^^)^

Data were entered into Microsoft Excel and analyzed using the (SPSS) version 22.0. Data were analyzed using descriptive and inferential statistics. Descriptive statistics involved calculation of absolute and relative distributions and statistical measures (mean and standard deviation), presented in table format. Inferential statistics were used to investigate associations between groups; significant differences were detected using the Mann-Whitney test and the Spearman correlation coefficient, with the level of significance set at 5%. Spearman ρ coefficient ranges from −1 to 1. The closer the value is to these extremes, the higher the association between variables. Correlations with a negative signal indicate the variables move in the opposite direction, *i.e.*, higher categories of one variable are associated with lower categories of the other variable.

This study was approved by the *Comitê de Ética em Pesquisa com Seres Humanos do Centro de Ciências da Saúde, Universidade Federal de Pernambuco* [Ethics Committe for Reserach with Humans] (opinion nº 1.478.011; CAAE: 51606015.8.0000.5208). Study participants signed an Informed Consent Form (version for participants aged over 18 years).

## RESULTS

This study comprised 271 undergraduate students (62% of them females) aged 18 to 45 years (mean age, 21.2±3.59; median age, 20 years).

Socioeconomic characteristics were as follows: most students (43.9%) belonged to the relatively lower class (class E) followed by classes C (24.4%) and D (28%). Classes A and B were less represented (2.2% and 0.7%, respectively). Household income data were lacking for two participants; these could therefore not be included in any of the classes, but were not excluded from the analysis, since lack of these data was not expected to compromise the overall purpose of the study.

Student distribution was not uniform given the offering of licentiate and bachelor degrees in Physical Education. Distribution was as follows: 47.2% Physical Education; 23.2% Nursing; 15.5% Nutrition; 14%, Biological Sciences.

Eating disorder and bulimia nervosa symptoms were identified in 7.4% (n=20) and 29.1% of students; EAT-26 and BITE respectively), with 24.7% (n=67) and 4.4% (n=12) of students with bulimia nervosa symptoms achieving medium and high BITE scores, respectively. Bulimic Investigatory Test of Edinburgh severity scale scores were non-significant in 92.6%, clinically significant in 6.3% (n=17) and highly severe in 1.1% (n=3) of participants.

As regards the risk of suicide according to MINI, 86.3% of participants were considered not to be risk of suicide, and 13.6% (n=37) scored between 1 and ≥10; of these, 7.4% (n=20) were thought to be at low, 0.7% (n=2) at moderate and 5.5% (n=15) at high risk of suicide.

Symptoms of major depression (HAM-D) were detected in 17.3% (n=47) of participants. Prevalence data and student distribution according to EAT-26, BITE symptom scale, BITE severity scale, HAM-D and MINI are shown in table 1.

**Table 1:** Student distribution according the different scales employed

		n (%)
EAT-26		
	No symptoms or indications of ED	251 (92.6)
	Presence of symptoms or indications of ED	20 (7.4)
BITE symptoms		
	Compulsive eating behavior	12 (4.4)
	Unusual eating behavior	67 (24.7)
	Normal eating behavior	192 (70.8)
BITE severity		
	High degree of severity	3 (1.1)
	Clinically significant	17 (6.3)
	Not severe	251 (92.6)
HAM-D		
	Lack of depressive symptoms	224 (82.7)
	Presence of depressive symptoms	47 (17.3)
MINI		
	No risk of suicide	234 (86.3)
	Low risk of suicide	20 (7.4)
	Moderate risk of suicide	2 (0.7)
	High risk of suicide	15 (5.5)

EAT: Eating Attitudes Test; ED: eating disorder; BITE: Bulimic Investigatory Test of Edinburgh; HAM-D: Hamilton Scale – Depression; MINI: Mini International Neuropsychiatric Interview.

Data association using the Spearman correlation coefficient revealed significant associations between the most important variables analyzed in this study; the association of MINI (risk of suicide) and EAT-26 (ED symptoms) scores yielded a positive correlation coefficient of 0.20 (p=0.001).

The risk of suicide also differed significantly between students with symptoms of bulimia nervosa in both BITE scales (symptom and severity) compared to students scoring zero in the BITE scale (p=0.001; p<0.001, respectively). The risk of suicide (MINI) was also positively correlated with symptoms of major depression (HAM-D) (correlation coefficient, 0.36; p<0.001). Correlations between variables according to clinical findings are described in table 2.

**Table 2:** Correlations between variables according to clinical findings

		MINI	HAM-D	EAT-26	BITE symptoms	BITE severity
MINI						
	Spearman	1.00	0.36	0.20	0.254	0.275
	p value*	–	<0.001	0.001	<0.001	<0.001
HAM-D						
	Spearman	–	1.00	0.378	0.391	0.355
	p value*	–	–	<0.001	<0.001	<0.001
EAT-26						
	Spearman	–	–	1.000	0.418	0.277
	p value*	–	–	–	<0.001	<0.001
BITE symptoms						
	Spearman	–	–	–	1.00	0.599
	p value*	–	–	–	–	<0.001
BITE severity						
	Spearman	–	–	–	–	1.000
	p value*	–	–	–	–	–

* Obtained via the Spearman correlation coefficient.

MINI: Mini International Neuropsychiatric Interview; HAM-D: Hamilton Scale – Depression; EAT: Eating Attitudes Test; BITE: Bulimic Investigatory Test of Edinburgh.

Association of variables according to sex revealed higher risk of eating disorders symptom development among women (p<0.001; Table 3).

**Table 3:** Results of association between variables according to sex

Instruments	Sex				p value*
	Female (n=168)		Male (n=103)		
	Mean	Standard deviation	Mean	Standard deviation	
BITE severity	1.83	2.51	1.69	2.05	0.938
BITE symptoms	8.22	5.48	6.83	4.96	0.028
EAT-26	12.74	7.87	9.82	5.99	<0.001
MINI	0.87	3.43	1.82	6.04	0.874
HAM-D	6.85	5.59	6.13	5.00	0.426

* Obtained via the Mann Whitney test.

BITE: Bulimic Investigatory Test of Edinburgh; EAT: Eating Attitudes Test; MINI: Mini International Neuropsychiatric Interview; HAM-D: Hamilton Scale – Depression.

## DISCUSSION

This study set out to identify eating disorders symptoms and investigate associations between eating disorders and the risk of suicide and depressive symptoms in students taking undergraduate courses in health-related areas.

Symptoms of eating disorders were detected in 7.4% of students, as reported in previous studies with similar students populations.^(^^7^^,^^20^^)^ Eating disorders are associated with biopsychosocial losses, with high morbidity and mortality rates. Some studies showed increasing incidence of eating disorders among undergraduate students, particularly those pursuing health careers, in which appearance is highly rated, such as Nutrition, Medicine, Nursing and Physical Education.^(^^7^^,^^20^^–^^23^^)^

However, a prevalence study conducted with students enrolled in undergraduate health-related courses in the state of São Paulo and using the same instrument (EAT-26) reported eating disorders symptoms in 26% of students.^(^^22^^)^ This may have reflected sociocultural differences between the Northeast and Southeast regions. Nonetheless, findings of that investigation are in keeping with findings derived from the pilot sample in this study.

Most of the 271 participants in this study were women. Female individuals had the highest percentage of eating disorders symptoms, as reported elsewhere.^(^^2^^,^^7^^,^^8^^,^^20^^)^ Female individuals are more prone to psychiatric disorders, such as eating disorders, given the high levels of pressure exerted on this sub-population by the media and society. Psychological, social and family-related factors contribute to the ideation of a perfect body and shape imposed by society.^(^^3^^,^^4^^,^^21^^,^^22^^)^ The desire to lose weight and the constant fear of weight gain are the major drivers of food-related concerns and adoption of self-guided dietary and physical exercise programs aimed to achieve socially perceivable weight loss. Anxiety and body-image disorders are major factors leading to eating disorders development.^(^^24^^)^

This study revealed a relatively concerning frequency rates of suicide risk in the population studied, supporting findings of previous studies.^(^^8^^,^^25^^,^^26^^)^ Death by suicide has increased significantly over the last decades, as have suicidal attempts and ideation.^(^^23^^)^ The risk of suicide is directly proportional to suicidal ideation, since the stronger the desire to die, the stronger the feelings of hopelessness and discontentment with life and the higher the lethality of the selected method, the greater the chances of suicidal behavior development.^(^^10^^)^ It is an established fact that no single factor can be held responsible for suicidal attempts or suicide itself. Suicide is a complex, multidimensional and multifactorial phenomenon involving several major risk factors, such as previous suicide attempts, genetic and social factors and psychiatric disorders, with particular emphasis on positive major depression symptoms and eating disorders.^(^^26^^–^^28^^)^

Almost one-fifth of participants in this sample had depression symptoms, as previously reported in undergraduate student and adolescent populations.^(^^8^^,^^28^^,^^29^^)^ According to the World Health Organization (WHO), depression is a major public health concern. Although depression may present as many episodes or occur once in a lifetime, it is thought to be a chronic problem associated with other health conditions, such as diabetes and heart failure. Likewise, suicide has been attracting increasing attention in public health settings and various research areas worldwide.^(^^30^^)^

As expected, this study revealed a positive correlation between depressive symptoms and risk of suicide. In fact, depression is so closely related to suicide that some authors describe suicide as an exclusive consequence, or even a symptom of depression.^(^^30^^–^^32^^)^ Also, suicidal ideation is often thought of as a typical symptom of depressive disorder.^(^^32^^)^ Growing prevalence of depression and risk of suicide have been reported in recent studies. Such findings hint at the alarming figures in higher education settings, particularly in health-related areas, with higher prevalence among students in the first years of university.^(^^31^^,^^33^^)^

As regards the risk of suicide and eating disorders symptoms, this study revealed significant associations in the population studied according to both screening tests (EAT-26 and BITE), with stronger associations in individuals with symptoms of BN compared to those scoring positive on EAT-26.

It is well established that suicide rates are higher among anorexia nervosa compared to bulimia nervosa patients,^(^^1^^,^^3^^,^^20^^)^ considering that anorexic individuals commit suicide less frequently, although they tend to progress to death more often due to anorexia nervosa-related systemic complications. Bulimic patients tend to commit suicide more often due to the typical impulsiveness associated with the condition; however, attempts are largely unsuccessful. Among other factors, this may explain the correlation between both BITE scales (symptoms and severity) and the risk of suicide in this study. This relation is even closer among individuals with positive BITE severity scale scores (p<0.001). Patients with bulimia nervosa avoid highly lethal methods and attempt suicide by substance inhalation, drug overdose, or other methods.^(^^32^^,^^33^^)^ This is not to say the risk of suicide is limited to eating disorders patients; rather, suicide is a major cause of mortality and morbidity among adolescents and young adults, since these stages of life are characterized by increased sensitiveness, including adoption of risky behaviors. Suicide risk and ideation may thus be related to other psychosocial factors and are multifactorial in nature.^(^^9^^)^

Eating disorders apparently increase the risk of suicide compared to most other psychiatric disorders; anorexia nervosa- and bulimia nervosa-related suicide rates are high and, among several factors, psychiatric disorders and drug addiction are the ones that stand out most.^(^^8^^)^

Research focusing on suicidal behavior have been routinely conducted and revealed several factors clearly associated with the risk of suicide, but no convincing theoretical basis to explain such findings. In most cases, these factors can be understood as mere correlations.^(^^31^^)^

Limitations of this study are related to study design, *i.e.,* single-center study based on data collected from one specific higher education institution and hence excluding other undergradute students in the same region. Also, potential sociocultural differences between regions preclude the generalization of findings. Lack of proper instruments to screen for eating disorders at the population level may interfere with diagnosis and treatment of the condition, introducing yet another constraint in eating disorders-related research. This was a risk assessment study involving non-diagnosed individuals; still, important associations were found. Larger studies are therefore warranted for external validity and comorbidity assessment.

## CONCLUSION

Undegraduate students with symptoms of eating disorders or depression were thought to be at higher risk of suicide risks. Identification of risk and protection factors must be followed by theories allowing for wide integration of new findings into the existing body of theoretical knowledge aimed to understand human behavior. Hence, undergraduate students with symptoms of major depression and eating disorders should be identified to mitigate suicide risks.
