# Supplementary material for: Association between eating disorders, suicide and depressive symptoms in undergraduate students of health-related courses
Source: Einstein (Sao Paulo). 2019 Dec 10;18:eAO4908. doi: 10.31744/einstein_journal/2020AO4908 (PMC6910065; doi:10.31744/einstein_journal/2020AO4908)
Supplement: Translation

# Translation: Associação entre transtornos alimentares, suicídio e sintomas depressivos em universitários de cursos de saúde

**Authors:** Vanigleidson Silva do Nascimento, Alisson Vinicius dos Santos, Suammy Barros Arruda, Gabriela Avelino da Silva, Joanna D'arc de Souza Cintra, Tiago Coimbra Costa Pinto, Rosana Christine Cavalcanti Ximenes

**Affiliations:** 1 Universidade Federal de Pernambuco, Recife, PE, Brasil

**Keywords:** Transtornos da alimentação e da ingestão de alimentos, Depressão, Suicídio, Estudantes de ciências da saúde

## Abstract

**Objetivo:**

Identificar sintomas de transtornos alimentares e possíveis associações com o risco de suicídio e sintomas depressivos em universitários de cursos de saúde.

**Métodos:**

Neste estudo de corte transversal, foram avaliados 271 estudantes. Foram utilizados os instrumentos Teste de Atitudes Alimentares e *Bulimic Investigatory Test of Edinburgh*, para identificação de sintomas de transtornos alimentares. Para o rastreamento de sintomas depressivos, foi usado o questionário de autoavaliação da Escala de Hamilton − Depressão, e o *Mini International Neuropsychiatric Interview*, para identificação do risco de suicídio. Todos os participantes responderam um questionário contendo informações sociodemográficas, para classificação econômica da amostra.

**Resultados:**

A frequência para sintomas de transtornos alimentares foi de 7,4% e de 29,1% para sintomas de bulimia nervosa. Cerca de 17,3% foram sintomáticos para depressão maior, e 13,6% tinham algum grau de risco de suicídio; destes, 7,4% foram considerados com risco de suicídio leve, 0,7% com risco moderado e 5,5% com risco alto de suicídio. Houve correlação entre risco para transtornos alimentares e risco de suicídio (p<0,001).

**Conclusão:**

Universitários com risco para os transtornos alimentares, bem como os que possuem sintomatologia sugestiva para depressão têm maior probabilidade de desenvolver o risco de suicídio.

## INTRODUÇÃO

A imposição e a estigmatização dos padrões de beleza podem levar à rejeição de obesos ou indivíduos com sobrepeso. Estas são razões que podem gerar sentimentos de desprezo e descontentamento com o próprio corpo, surgindo o medo de engordar.^(^^1^^)^ Os comportamentos alimentares alterados e as práticas de controle de peso estão associados com a insatisfação pessoal e a baixa autoestima, que podem se expressar na perda de peso.^(^^2^^)^

A mídia tem sido o mais poderoso aliado na missão de estabelecer que o protótipo de beleza e saúde é um corpo magro ou até mesmo esquelético. Isso tem aumentado a valorização do peso e da forma corporal, principalmente no público feminino. Consequentemente, os indivíduos são levados a realizarem práticas que podem comprometer a saúde de forma severa, como exercícios físicos em excesso e dietas radicais, com a finalidade de atingir o ideal de corpo.^(^^3^^)^

A frequente descrição da insatisfação corporal e das altas taxas de suicídio nos últimos anos fez com que um segmento da literatura científica entendesse os transtornos alimentares como parte dos transtornos psiquiátricos. Até então, porém, não há causa preestabelecida.^(^^3^^–^^5^^)^

Os transtornos alimentares são transtornos psiquiátricos graves, de origem multifatorial, caracterizados por alterações no comportamento alimentar, tendo como principais subtipos a anorexia nervosa e a bulimia nervosa.^(^^1^^,^^3^^,^^4^^)^ Apresentam elevados índices de mortalidade, incapacidade, morbidade física e psicológica, além de associação com redução da qualidade de vida.^(^^6^^,^^7^^)^ Assim, necessitam de enorme atenção das políticas públicas de saúde, já que pessoas escondem a doença e evitam pedir ajuda profissional.^(^^1^^)^

As mudanças físicas e psicológicas normais na adolescência podem fazer com que se desenvolvam indicadores de transtornos alimentares desde esta fase, em especial da anorexia nervosa e da bulimia nervosa, em razão da imaturidade cognitiva para se adaptar às mudanças fisiológicas do organismo.^(^^8^^)^ Muitos casos diagnosticados estão intimamente ligados a altas taxas de mortalidade – 15% desses pacientes vão a óbito.^(^^2^^,^^7^^)^ Dentre outros fatores, a presença de transtornos alimentares pode contribuir para o risco de suicídio,^(^^8^^)^ uma das principais causas de morbidade e mortalidade entre os adolescentes e jovens adultos, pois esses períodos são caracterizados por ascensão de riscos e comportamentos imprudentes.^(^^9^^)^

Diversas associações envolvendo o risco de suicídio foram publicadas na literatura, como histórico familiar de suicídio, uso de substâncias químicas, maus tratos na infância e transtornos psiquiátricos, com destaque para o transtorno de humor, transtornos alimentares (principalmente anorexia nervosa e bulimia nervosa) e sintomatologia positiva para depressão maior. Todavia, dentre todos os transtornos psiquiátricos, os transtornos alimentares parecem levar a um maior risco de suicídio quando comparados a outros transtornos de cunho psiquiátrico.^(^^10^^)^ As prevalências de depressão são mais altas em todos os tipos de transtornos alimentares do que na população em geral.^(^^11^^)^

Há estimativas de que 15% a 25% dos universitários apresentam algum tipo de transtorno psiquiátrico no decorrer de sua formação acadêmica, especialmente aquelas da área de saúde.^(^^9^^,^^11^^)^ No entanto, poucos foram os estudos que consideram esse público nos levantamentos epidemiológicos.

A presença de transtornos alimentares em universitários pode estar relacionada com a depressão, e esses fatores podem aumentar o risco de suicídio nessa população.

## OBJETIVO

Identificar sintomas de transtornos alimentares e determinar possíveis associações com o risco de suicídio e sintomas depressivos em universitários de cursos de saúde.

## MÉTODOS

Trata-se de estudo epidemiológico de corte transversal desenvolvido em universidade pública do interior de Pernambuco. A amostra deste estudo foi composta por universitários de ambos os sexos de cursos de Saúde com idade igual ou superior a 18 anos. O total de universitários do *campus* era de 1.525 estudantes. Os cursos oferecidos pela instituição eram Enfermagem, Nutrição, licenciatura em Ciências Biológicas e licenciatura e bacharelado em Educação Física. O curso de saúde coletiva também era ofertado, mas ainda possuía o número de turmas bastante reduzido, inviabilizando a representatividade deste curso na pesquisa.

Para o cálculo amostral de uma população representativa do centro acadêmico, utilizou-se o programa Epi Info™. Além disso, foi utilizada a prevalência encontrada numa amostra piloto. Esta amostra foi selecionada apenas para testar o método proposto, verificar se os instrumentos estavam adequados, treinar o pesquisador e obter uma prevalência inicial que seria usada para o cálculo da amostra final. As avaliações feitas nesta etapa não foram consideradas para o estudo principal, por se tratar de um teste. Sendo assim, com a proporção esperada de 33,1% de sintomas de transtornos alimentares obtida na amostra piloto realizada com 30 universitários, erro de 5,0% e confiabilidade de 95,0%, chegou-se em amostra de 278 estudantes. No entanto, houve perda de 7 avaliações por desistência, e a amostra final foi de 271 universitários.

A seleção dos alunos foi feita de forma aleatória, por sorteio, mediante a lista de frequência fornecida pela universidade. Os alunos sorteados foram contatados para pesquisa nas salas de aula. Quando o estudante sorteado não estava presente ou se recusava a participar, o aluno subsequente era convocado.

A coleta de dados foi realizada no período de 3 meses, nas salas de aula da universidade, no horário do intervalo das aulas dos estudantes. Os graduandos participaram da coleta em seu turno de frequência. Com a finalidade de descrever o perfil socioeconômico da amostra pesquisada, foi aplicado questionário sociodemográfico.^(^^12^^)^ Os seguintes dados foram obtidos: idade, sexo, se tinha irmãos, ordem de nascimento, número de cômodos da residência e número de pessoas que moravam com o jovem. Além disso, o questionário continha dados de classificação econômica, de acordo com os Critérios de Classificação Econômica do Brasil, de 2015, da Associação Brasileira das Empresas de Pesquisa (ABEP). Nesta classificação, o estrato socioeconômico A corresponde à renda média familiar de R$ 20.888,00 ou valores superiores; B1, a partir de R$ 9.254,00; B2, acima de R$ 4.852,00; C1, de R$ 2.705,00; C2, de R$ 1.625; D, de R$ 768,00 e classe E, abaixo deste valor.

Para a identificação de sintomas de transtornos alimentares foi usado o Teste de Atitudes Alimentares (EAT-26 - *Eating Attitudes Test-26*).^(^^13^^,^^14^^)^ Cada questão, dividida em três escalas do tipo Likert, apresenta seis opções de resposta, conferindo-se pontos de zero a 3, dependendo da escolha do participante. Por fim, as pontuações de cada item são somadas e obtém-se um escore final. Os participantes que atingiram pontuação ≥21 pontos foram considerados de risco para o desenvolvimento de transtornos alimentares e os com escore de zero a 20 pontos, isentos de risco. Não é possível estabelecer o diagnóstico por meio do teste, mas ele detecta casos clínicos em populações de alto risco e identifica indivíduos com preocupações anormais em relação à alimentação e ao peso.^(^^2^^,^^8^^)^

Para detecção e a avaliação da gravidade da bulimia nervosa, foi aplicado o *Bulimic Investigatory Test of Edinburgh* (BITE),^(^^15^^,^^16^^)^ composto por duas escalas: uma de sintomas (30 itens sim/não, com escore variando de zero a 30) e outra de gravidade (três itens dimensionais). Esses dois escores podem ser adicionados para produzir um escore total. Na escala de sintomas, um escore elevado (≥20) pode sugerir padrão alimentar muito perturbado e a presença de compulsão alimentar; escores médios (entre 10 e 19) sugerem padrão alimentar não usual, necessitando avaliação por uma entrevista clínica, e escores abaixo de 10 estão dentro dos limites de normalidade. Na escala de gravidade, pontuação ≥5 é considerada clinicamente significativa, e ≥10 indica elevado grau de gravidade.

A *Mini International Neuropsychiatric Interview* (MINI), na versão brasileira 5.0.0, Módulo C – Risco de Suicídio, corresponde a uma entrevista que compreende 19 módulos, que avaliam 17 transtornos do eixo I do Manual de Diagnóstico e Estatística dos Transtornos Mentais – Quarta Edição™ (DSM-IV™), risco de suicídio e transtorno de personalidade antissocial.^(^^17^^)^ No presente estudo, foi avaliado apenas o módulo C – Risco de Suicídio. Os universitários com pontuação zero foram classificados como “sem risco de suicídio”, de 1 a 5 pontos, como “baixo risco de suicídio”, de 6 a 9 pontos, como “moderado risco de suicídio”, e de 10 pontos ou mais, como “alto risco de suicídio”.

O questionário de autoavaliação da Escala de Hamilton − Depressão (HAM-D - *Hamilton Depression Rating Scale*), desenvolvido na Universidade de Londres a partir da Escala de Hamilton − Depressão,^(^^18^^)^ foi traduzido para o português^(^^19^^)^ e validado na população brasileira. Quando selecionado um ponto de corte de 10, as informações obtidas pelo HAM-D foram bastante precisas em discriminar pacientes deprimidos de um grupo controle normais.^(^^18^^)^

Os dados foram registrados em banco no Microsoft Excel e avaliados no programa (SPSS) versão 22.0. Para a análise dos dados, foram utilizadas técnicas de estatística descritiva e inferencial. A estatística descritiva envolveu a obtenção das distribuições absolutas, relativas e das medidas estatísticas: média e desvio padrão, apresentadas sob a forma de tabelas. As técnicas de estatística inferencial foram utilizadas para analisar a associação entre os grupos, observando a presença de diferenças significantes, por meio do teste de Mann-Whitney e do coeficiente de correlação de Spearman, considerando-se sempre nível de significância de 5%. O coeficiente ρ de Spearman varia entre −1 e 1. Quanto mais próximo estiver destes extremos, maior a associação entre as variáveis. O sinal negativo da correlação significa que as variáveis variam em sentido contrário, isto é, as categorias mais elevadas de uma variável estão associadas a categorias mais baixas da outra variável.

Este estudo foi aprovado pelo Comitê de Ética em Pesquisa com Seres Humanos do Centro de Ciências da Saúde da Universidade Federal de Pernambuco (parecer 1.478.011; CAAE: 51606015.8.0000.5208). Todos os participantes do estudo assinaram o Termo de Consentimento Livre e Esclarecido (TCLE) em sua versão para maiores de 18 anos.

## RESULTADOS

O presente estudo contou com amostra de 271 universitários (62% eram do sexo feminino). A idade dos participantes do estudo variou entre 18 e 45 anos, tendo como média 21,2±3,59 anos e mediana de 20 anos.

Em relação às características socioeconômicas, a maioria dos estudantes (43,9%) era de classe relativamente mais baixa, correspondente à classe E, seguida das classes C (24,4%) e D (28%). Em menor percentual, as classes A e B, tiveram 2,2% e 0,7%, respectivamente. Apenas dois participantes não informaram seu nível de renda, não sendo possível classificá-los em nenhuma das classes citadas. Esses indivíduos não foram excluídos da pesquisa, uma vez que a ausência dessas informações não comprometeu o objetivo geral do estudo.

A distribuição dos estudantes não foi uniforme devido ao fato de o curso de educação física ser oferecido nas modalidades licenciatura e bacharelado. Assim, foi obtido o seguinte percentual: 47,2% para educação física; 23,2% para enfermagem; 15,5% para nutrição e 14% para ciências biológicas.

A frequência de universitários com sintomas de transtornos alimentares, segundo o EAT-26, foi de 7,4% (n=20). Conforme o BITE, 29,1% dos pesquisados tinham sintomas de bulimia nervosa, dos quais 24,7% (n=67) obtiveram escore médio; enquanto 4,4% (n=12) tinham escore elevado. Na escala de gravidade BITE, 92,6% dos pesquisados tiveram escore não significativo, sendo 6,3% (n=17) clinicamente significativo, e 1,1% (n=3) obtiveram escore de gravidade elevada.

Com relação ao risco de suicídio, segundo a MINI, 86,3% dos pesquisados foram considerados sem risco de suicídio, e 13,6% (n=37) obtiveram escores entre 1 e ≥10; destes, 7,4% (n=20) foram considerados com risco de suicídio leve; 0,7% (n=2) com risco moderado e 5,5% (n=15) com risco alto de suicídio.

Constatou-se que 17,3% (n=47) tinham sintomatologia de depressão maior, segundo o HAM-D. Na tabela 1 estão descritos os principais resultados das prevalências obtidas no estudo, bem como a distribuição dos universitários da pesquisa, de acordo o EAT-26, o BITE sintomatologia, o BITE gravidade, o HAM-D e a MINI.

**Tabela 1:** Universitários da amostra distribuídos de acordo as escalas utilizadas

		n (%)
EAT-26		
	Sem sintomas ou indicativo de TA	251 (92,6)
	Presença de sintomas ou indicativo de TA	20 (7,4)
BITE sintomatologia		
	Presença de comportamento alimentar compulsivo	12 (4,4)
	Comportamento alimentar não usual	67 (24,7)
	Comportamento alimentar normal	192 (70,8)
BITE gravidade		
	Elevado grau de gravidade	3 (1,1)
	Clinicamente significativo	17 (6,3)
	Ausência de gravidade	251 (92,6)
HAM-D		
	Ausência de sintomas depressivos	224 (82,7)
	Presença de sintomas depressivos	47 (17,3)
MINI		
	Risco de suicídio ausente	234 (86,3)
	Risco de suicídio leve	20 (7,4)
	Risco de suicídio moderado	2 (0,7)
	Risco de suicídio alto	15 (5,5)

EAT: Teste de Atitudes Alimentares; TA; transtorno alimentar; BITE: *Bulimic Investigatory Test of Edinburgh*; HAM-D: Escala de Hamilton − Depressão; MINI: *Mini International Neuropsychiatric Interview*.

Após a associação dos dados, com a utilização do coeficiente de correlação de Spearman, foi possível observar que houve significância estatística entre as principais variáveis analisadas, de modo que, ao se associarem os escores da MINI (risco de suicídio) e do EAT-26 (sintomatologia de transtornos alimentares), encontrou-se coeficiente de correlação positiva de 0,20 (p=0,001).

Houve também diferença estatística para o risco de suicídio entre universitários com sintomas de bulimia nervosa em ambas as escalas BITE (sintomatologia e gravidade) em relação àqueles que não pontuaram na escala (p=0,001; p<0,001, respectivamente). Houve também correlação positiva entre o risco de suicídio (MINI) e os sintomas de depressão maior (HAM-D), e o coeficiente de correlação para essa associação foi de 0,36 (p<0,001). Os resultados da correlação entre as variáveis, segundo os achados clínicos, estão descritos na tabela 2.

**Tabela 2:** Correlação entre as variáveis, segundo os achados clínicos

		MINI	HAM-D	EAT-26	BITE sintomatologia	BITE gravidade
MINI						
	Spearman	1,00	0,36	0,20	0,254	0,275
	Valor de p*	–	<0,001	0,001	<0,001	<0,001
HAM-D						
	Spearman	–	1,00	0,378	0,391	0,355
	Valor de p*	–	–	<0,001	<0,001	<0,001
EAT-26						
	Spearman	–	–	1,000	0,418	0,277
	Valor de p*	–	–	–	<0,001	<0,001
BITE sintomatologia						
	Spearman	–	–	–	1,00	0,599
	Valor de p*	–	–	–	–	<0,001
BITE gravidade						
	Spearman	–	–	–	–	1,000
	Valor de p*	–	–	–	–	–

* Obtido por meio do coeficiente de correlação de Spearman.

MINI: *Mini International Neuropsychiatric Interview*; HAM-D: Escala de Hamilton − Depressão; EAT: *Eating Attitudes Test*; BITE: *Bulimic Investigatory Test of Edinburgh*.

Ao associar as variáveis de acordo com o sexo, mulheres tiveram mais chances de desenvolver sintomatologia para TA (p<0,001). Na tabela 3 está descrita essa associação.

**Tabela 3:** Resultado da associação entre as variáveis segundo o sexo

Instrumentos	Sexo				Valor de p*
	Feminino (n=168)		Masculino (n=103)		
	Média	Desvio padrão	Média	Desvio padrão	
BITE gravidade	1,83	2,51	1,69	2,05	0,938
BITE sintomatologia	8,22	5,48	6,83	4,96	0,028
EAT-26	12,74	7,87	9,82	5,99	<0,001
MINI	0,87	3,43	1,82	6,04	0,874
HAM-D	6,85	5,59	6,13	5,00	0,426

* Obtido por meio do teste de Mann Whitney.

BITE: *Bulimic Investigatory Test of Edinburgh*; EAT: *Eating Attitudes Test*; MINI: *Mini International Neuropsychiatric Interview*; HAM-D: Escala de Hamilton − Depressão.

## DISCUSSÃO

Este estudo buscou identificar sintomas de transtornos alimentares e averiguar se esse risco está associado com o risco de suicídio e os sintomas depressivos em universitários de cursos de saúde.

Foi encontrada frequência de 7,4% de universitários com sintomas de transtornos alimentares, corroborando achados de outros estudos com acadêmicos da área da saúde.^(^^7^^,^^20^^)^ Os transtornos alimentares são distúrbios que levam a prejuízos biopsicossociais com elevada taxa de morbimortalidade na população. Estudos mostram o quanto a incidência de transtornos alimentares têm aumentado na população universitária. Tal achado é ainda maior quando se trata de cursos de saúde, principalmente naqueles em que a aparência é tida como importante − dentre eles, destacam-se os cursos de nutrição, medicina, enfermagem e educação física.^(^^7^^,^^20^^–^^23^^)^

Entretanto, estudo de prevalência realizado no estado de São Paulo com acadêmicos de cursos de saúde encontrou frequência de 26% de universitários com sintomas de transtornos alimentares usando o mesmo instrumento EAT-26.^(^^22^^)^ Isso pode ter acontecido devido às diferenças socioculturais existentes entre as Regiões Nordeste e Sudeste. Contudo, tal resultado está dentro do esperado, mediante achados da amostra piloto.

Dos 271 pesquisados, a maioria era do sexo feminino, e foram os indivíduos desse sexo que apresentaram maior percentual de sintomas de transtornos alimentares, corroborando outros achados em estudos com universitários.^(^^2^^,^^7^^,^^8^^,^^20^^)^ Indivíduos do sexo feminino são mais propensos a desenvolverem diversos transtornos psiquiátricos, como os transtornos alimentares, uma vez que, desde a adolescência, são enormes as pressões midiáticas e sociais sobre essa população. Fatores psicológicos, sociais e familiares colaboram com a ideação do corpo e da forma perfeitos impostos pela sociedade.^(^^3^^,^^4^^,^^21^^,^^22^^)^ O desejo de emagrecer e o persistente medo de engordar são os principais responsáveis por desencadear uma preocupação com os alimentos e as mais diversas maneiras de fazer dieta sem acompanhamento profissional, associadas à prática de exercícios físicos, na intenção de que haja perda de peso perceptível pela sociedade. Ainda, existem outros fatores, como o transtorno de ansiedade e o distúrbio da autoimagem corporal, que são uns dos pilares que levam ao surgimento de transtornos alimentares.^(^^24^^)^

O presente estudo encontrou frequência relativamente preocupante para o risco de suicídio na população estudada, e tais resultados corroboram os achados de estudos anteriores.^(^^8^^,^^25^^,^^26^^)^ As mortes por suicídio vêm crescendo significativamente nas últimas décadas, bem como a tentativa e a ideação suicida.^(^^23^^)^ O risco de suicídio é diretamente proporcional à ideação suicida, pois quanto maiores o desejo de morte e o poder de letalidade do método de escolha, bem como o sentimento de desesperança e o descontentamento com a vida, maiores as chances de um indivíduo desenvolver o comportamento suicida.^(^^10^^)^ Existe o consenso de que não há apenas um fator que responda à tentativa e nem ao suicídio propriamente dito. Portanto, o suicídio consiste em um fenômeno complexo, multidimensional e multifatorial, tendo como principais fatores de risco tentativas prévias de suicídio, fatores genéticos e sociais, e transtornos psiquiátricos (com destaque para a sintomatologia positiva para depressão maior e transtornos alimentares).^(^^26^^–^^28^^)^

Foi possível constatar também que quase um quinto dos pesquisados nesta amostra foram sintomáticos para depressão, corroborando outros estudos em populações de universitários e adolescentes.^(^^8^^,^^28^^,^^29^^)^ Segundo a Organização Mundial da Saúde (OMS), a depressão ocupa posições de destaque no quadro dos problemas de saúde pública. Embora ela possa ocorrer em diversos episódios ou uma vez só em toda a vida, é considerada um problema crônico e que traz mais afecções para a saúde humana do que problemas como diabetes ou insuficiência cardíaca. Simultaneamente, o suicídio também tem recebido destaque na saúde pública e despertado o interesse de pesquisadores em todo o mundo das mais diferentes áreas da ciência.^(^^30^^)^

No presente estudo, houve, como esperado, correlação positiva entre sintomatologia depressiva e risco de suicídio, pois a relação entre suicídio e depressão é tão estreita, que existem autores que consideram o suicídio como consequência exclusiva da depressão, ou ainda um sintoma da mesma.^(^^30^^–^^32^^)^ Além disso, a ideação suicida é frequentemente considerada um dos sintomas característicos do transtorno depressivo.^(^^32^^)^ A depressão e o risco de suicídio têm tido crescente prevalência nos estudos mais recentes sobre a temática. Tais achados mostram o quanto esse número é preocupante dentro das universidades, principalmente na área da saúde, com maior prevalência no ciclo básico dos mais diferentes cursos da referida área.^(^^31^^,^^33^^)^

Com relação à presença do risco de suicídio e sintomas de transtornos alimentares, foram observadas significantes associações na população estudada em ambos os testes de rastreamento EAT-26 e BITE, sendo possível notar que essa associação foi maior ainda naqueles indivíduos que apresentaram sintomas de bulimia nervosa quando comparados aos que pontuaram positivamente no EAT-26.

Está bem documentado que, em pacientes com anorexia nervosa, as taxas de suicídio são mais elevadas quando comparadas aos portadores de bulimia nervosa,^(^^1^^,^^3^^,^^20^^)^ visto que mesmo os anoréxicos tentando menos suicídio, eles morrem mais devido às complicações sistêmicas causadas pela anorexia nervosa. Já os bulímicos tentam mais suicídio, tendo em vista a impulsividade característica desse transtorno, porém essas tentativas geralmente não são efetivas. Esses são alguns dos fatores que podem explicar a correlação encontrada neste estudo entre ambas as escalas do BITE (sintomatologia e gravidade) e o risco de suicídio. Tal relação é ainda mais estreita naqueles indivíduos que pontuaram positivamente na escala de gravidade do BITE (p<0,001). Os pacientes com bulimia nervosa evitam métodos mais letais: tentam suicídio por inalação de substâncias e *overdose* de drogas entre outros.^(^^32^^,^^33^^)^ Isso não quer dizer que o risco de suicídio pode ser encontrado apenas em pacientes com transtornos alimentares, pois o suicídio é uma das principais causas de mortalidade e morbidade entre adolescentes e jovens adultos, já que esses períodos são caracterizados por sensibilidade aumentada, incluindo a assunção de riscos e comportamentos imprudentes, de modo que o risco e a ideação suicida podem estar relacionados a outros fatores psicossociais, tornando-o um problema multifatorial.^(^^9^^)^

Os transtornos alimentares parecem levar a um maior risco de suicídio quando comparados com a maioria de outros transtornos psiquiátricos, pois na anorexia nervosa e na bulimia nervosa as taxas de suicídio são elevadas e, dentre os mais diversos fatores associados, os que mais se destacam são os transtornos de origem psiquiátrica e dependência química.^(^^8^^)^

No campo da pesquisa sobre comportamento suicida, os estudos têm tido caráter rotineiro e produziram panorama de fatores claramente associados ao risco de suicídio, sem, no entanto, oferecer fundamentação teórica convincente e consistente para os achados. Estes fatores, na maioria das vezes, podem ser tomados apenas como correlações.^(^^31^^)^

As limitações deste estudo estão relacionadas ao fato de serem apresentados dados referentes a um centro específico de ensino superior, não podendo contemplar todos os universitários da região. Além disso, devem ser levadas em conta as diferenças socioculturais entre as regiões, fator esse que não permite generalizar nossos achados. Existe também dificuldade em pesquisas com transtornos alimentares para se encontrarem instrumentos adequados para o rastreio desses comportamentos nas populações, e isso pode dificultar o diagnóstico e o tratamento. Assim, sugerem-se estudos maiores, para que sejam possíveis a avaliação da validade externa e o exame das causas das comorbidades, uma vez que, mesmo se tratando apenas de avaliação de risco, foram encontradas associações importantes, ainda que em indivíduos não diagnosticados.

## CONCLUSÃO

Os universitários com sintomas de transtornos alimentares, bem como os que possuem sintomatologia de depressão, tinham maior probabilidade de desenvolver o risco de suicídio. A identificação de fatores de risco e de proteção deve ser seguida de teorias que possam, de forma abrangente, integrar os novos achados ao corpo de conhecimento teórico, que procura compreender o comportamento humano. Por conseguinte, sugere-se que universitários sintomáticos para depressão maior e transtornos alimentares sejam investigados, a fim de minimizar as chances de esses indivíduos desenvolverem o risco para o suicídio.
